# The Blind Man at Work

**Published:** 1923-04

**Authors:** 


### THE BLIND MAN AT WORK.

If there is one class of human beings more assured of the support and sympathy of us all—provided that our sluggish memories are constantly jogged—it is the class of those who enter upon their daily round always in total darkness. But our sympathy and support need guidance. When the blind beggar, groping his way along the street with his stick and his dog and his little tin can, receives the coins of the passers-by, the individual giver does himself credit. But it is discreditable to the community of which the blind man with his little tin can forms a part that the coin should have to be given in that way.

### The Bradford Royal Institution.

In Bradford, where Lord Onslow, acting for the Minister of Health the other day, opened new Workshops for the Blind, the blind beggar has disappeared from the streets. That is a really fine thing to record. It does not mean that he is in the workhouse! In fact, no blind person in Bradford, unless suffering from some serious ailment other than blindness, is left in the workhouse. The Bradford Royal Institution has long been recognised as the one which provides the most complete service for its own blind in the country. Its activities comprise employment and training—wages and allowances amounted in 1921 to £7,500 and sales to £23,000—two homes, a hostel for women workers, six home visitors, and social welfare work of all kinds. It has been built up entirely by public-spirited, self-sacrificing voluntary effort. The relations which exist between the Institution and the local Education, Municipal and Poor Law authorities are most cordial and helpful. Exchequer assistance is to some extent available. The Town Council propose, in the exercise of their duties under the Blind Persons Act, 1920, to utilise and support the splendid voluntary work that is being carried on by the Institution.

### Practical Forms of Help.

The first step in the direction of organised public help for our blind fellow-citizens must be the placing of the finances of such an institution as that at Bradford "beyond a peradventure." The debt on the new workshops must be cleared off at once. Subscriptions adequate for the maintenance in comfort of those past work, and for the social welfare of all, must be forthcoming. But it is the maintenance of the blind man and woman at work that should concern us most, and their wise and sympathetic training, with that end in view, in workshops and in their own homes. We must help the blind to overcome their disability, to forget their separation from their fellows, and to enter as far as possible into our common life as citizens. The most effective form of all assistance is that which leads up to and ensures a market for articles manufactured by the blind person under conditions which must always be difficult and uneconomic. It is a way in which local authorities and other public bodies as well as individuals can help. It is the way essentially in which lies the maintenance of the blind man's self-respect.

